# Topographic Electrophysiological Signatures of fMRI Resting State Networks

**DOI:** 10.1371/journal.pone.0012945

**Published:** 2010-09-22

**Authors:** Kay Jann, Mara Kottlow, Thomas Dierks, Chris Boesch, Thomas Koenig

**Affiliations:** 1 Department of Psychiatric Neurophysiology, University Hospital of Psychiatry and University of Bern, Bern, Switzerland; 2 Department of Clinical Research, AMSM, Inselspital and University of Bern, Bern, Switzerland; Freie Universitaet Berlin, Germany

## Abstract

**Background:**

fMRI Resting State Networks (RSNs) have gained importance in the present fMRI literature. Although their functional role is unquestioned and their physiological origin is nowadays widely accepted, little is known about their relationship to neuronal activity. The combined recording of EEG and fMRI allows the temporal correlation between fluctuations of the RSNs and the dynamics of EEG spectral amplitudes. So far, only relationships between several EEG frequency bands and some RSNs could be demonstrated, but no study accounted for the spatial distribution of frequency domain EEG.

**Methodology/Principal Findings:**

In the present study we report on the topographic association of EEG spectral fluctuations and RSN dynamics using EEG covariance mapping. All RSNs displayed significant covariance maps across a broad EEG frequency range. Cluster analysis of the found covariance maps revealed the common standard EEG frequency bands. We found significant differences between covariance maps of the different RSNs and these differences depended on the frequency band.

**Conclusions/Significance:**

Our data supports the physiological and neuronal origin of the RSNs and substantiates the assumption that the standard EEG frequency bands and their topographies can be seen as electrophysiological signatures of underlying distributed neuronal networks.

## Introduction

Most brain activity is covert and does not lead to any directly observable behavior. There is however abundant evidence showing that mental activity under no-task conditions is well structured and has defined population norms that vary systematically with development [1], [2]. Deviations from those norms are observed in many clinically relevant conditions including neurological and psychiatric disorders [3], varying states of consciousness [4], sleep [5] or under neurotropic medication [6]. Paralleling the EEG literature, in fMRI and PET so called resting state networks (RSNs) have been established recently [7], [8], [9]. These RSNs are defined by synchronized spontaneous low-frequency oscillations between the areas composing the specific RSN. Consistent RSNs have been described for visual, motor, auditory and attention networks [10], [11]. These RSNs are sensitive to task execution [12] and different neuropsychiatric diseases [13].

EEG and fMRI have a common origin [14], and both show a spontaneous structuring, but represent very different aspects of brain activity. Their relationship can thus yield insights not available from one modality alone. Interestingly, when investigating BOLD correlates of EEG rhythms [15], [16] these correlates resembled the RSNs, suggesting that the different RSNs assemble through synchronization of electric activity as measured by EEG [17], [18], [19]. Namely, BOLD correlates of electrical activity in the alpha (8–12Hz) and beta (17–23Hz) frequency band displayed striking similarity with two RSNs described in other publications [9], [20], suggesting that the RSNs may be organized by neuronal activity at specific frequencies. This hypothesis was recently extended [19] by directly correlating the temporal dynamics of EEG spectral fluctuations across all frequency bands with the temporal dynamics of different RSNs. Several RSNs had specific and unique correlation patterns across frequency bands, indicating a complex relationship between RSNs and EEG rhythms. In a recent study we could demonstrate that a further subdivision of the standard EEG alpha frequency band into a lower (8.5–10.5Hz) and an upper (10.5–12.5Hz) alpha band showed correlations in different RSNs [17].

However, still little is known about the scalp distribution of the EEG spectral correlates of fMRI RSNs. This is surprising, since the spatial configuration of scalp fields and spectral power is an important and sensitive marker for changes of brain functional state, on a local and global level. Deviations from the resting state during task execution or alterations caused by disease are often local, i.e at delimited scalp locations. Furthermore, within one EEG frequency band, several functionally different EEG rhythms may coexist that can be distinguished by their topography, and changes of the distribution of EEG rhythms are well-established markers for changes of brain state.

Therefore, the primary purpose of the current study was to explore the topographic distribution of the EEG spectral power fluctuations in association to different RSNs. Introducing the spatial dimension of EEG spectral fluctuations with regard to RSN dynamics might provide an important link between fMRI RSN literature and EEG literature, namely because for many of the cognitive domains that are associated with specific RSNs (e.g. attention, vision, motor system) it has also been shown that changes of activation induce consistent, and localized changes in EEG spectra. Furthermore, we expected that information about the topographic relation of EEG spectral fluctuations might help to partly resolve the issue of inconsistent and even contradicting results in the existing EEG-fMRI RSN literature (for an overview see [21]). While in previous studies the BOLD signal fluctuations in each voxel or of a whole RSN were explained by a single EEG feature (e.g. arbitrary single or few channels [15], [16], [22] or global features such as global field power [19] or global field synchronization [17]), we provide the relation of the variance of EEG spectral power at each electrode to the dynamics of different RSN using Covariance Mapping [23]. This yields the topographic distribution of the RSN-EEG frequency relationship. It is important to note here that despite the well-known difficulties of the EEG inverse problem that limits the 3D resolution of the data, EEG scalp distributions are very sensitive for even small changes in the arrangement of the generators and may therefore be more suitable to analyze RSNs than global or single electrode measures of EEG spectral fluctuations. In order to avoid possible problems with multiple testing when analyzing high-density EEG data, we consistently relied on global randomization statistics that do not inflate the alpha-error.

## Results

### ICA and group statistics of RSNs

**Figure 1:**
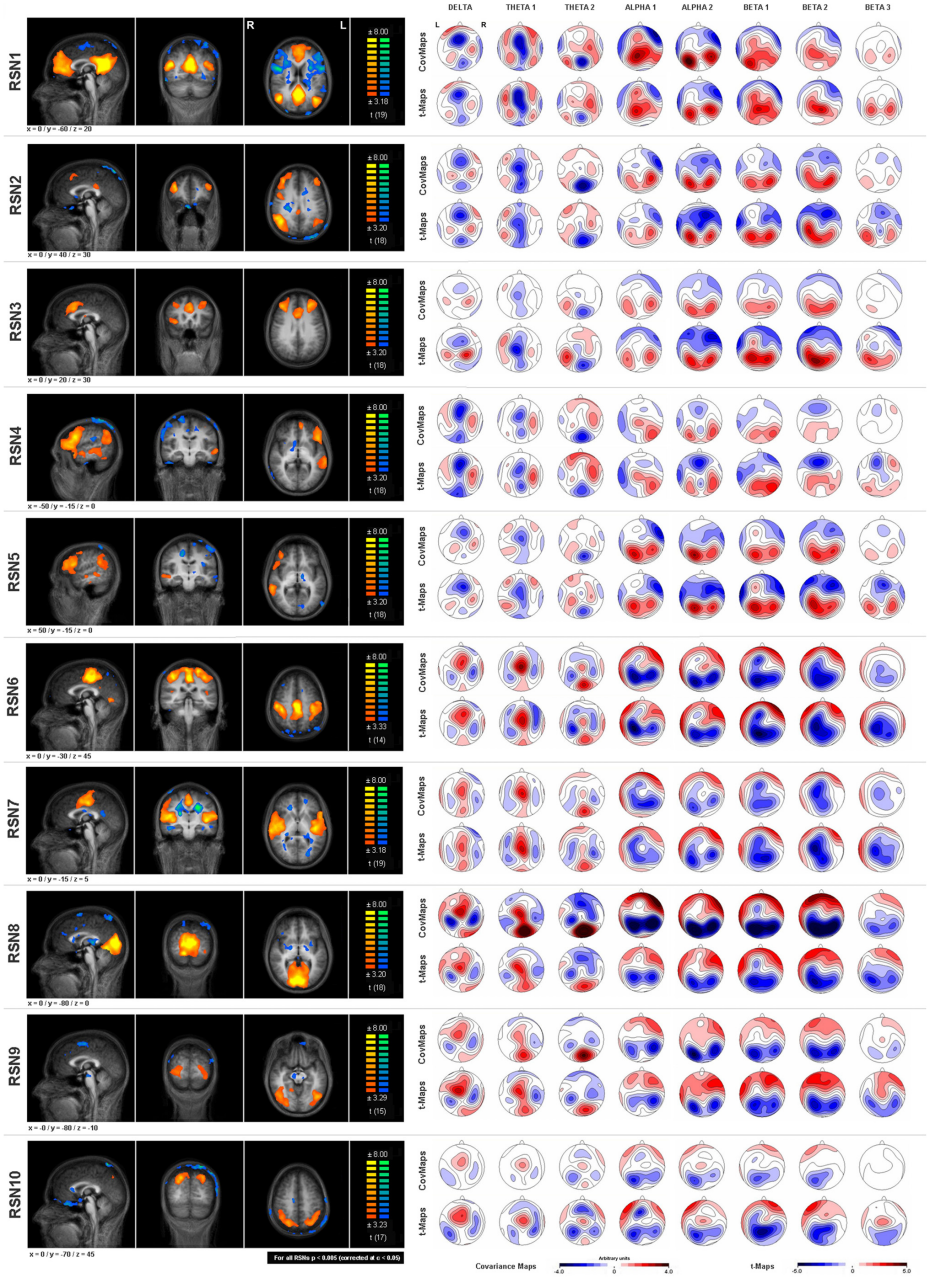
RSNs and their topographic EEG signatures. The left row shows sagittal, coronal and horizontal slices of the ten RSNs (p<0.005; corrected at α<0.05 / x, y & z coordinates are provided at the left bottom corner of each RSN). On the right side the covariance and t-maps for the 8 frequency bands are displayed. A positive covariance value (red) indicates that with increasing RSN activity there is a relative increase in spectral power at a given electrode in a given frequency band, while a negative value (blue) indicates a decrease in power when the RSN activity increases, and vice versa. [Note: MR images are in neuroradiological convention (left is right), EEG maps are not (left is left).]

From the thirty independent components (ICs) computed in each subject we selected those ten ICs whose maps were most similar to previously described RSNs [10], [11], [24] and were not related to motion artifacts or blood cycling [25]. However, we were not able to identify ICs for all targeted RSNs in all subjects, but a targeted RSN had a matching individual IC in 18.4±1.6 subjects in the average. Thus, in the computation of the group components (GCs) only individuals with an assigned IC for a given RSN were taken into account (Table 1 last column). The similarity means laid above 0.30, matching the values typically reported for the clustering of ICs [26]. The ten GC representing the RSNs are displayed in Figure 1 (left row).

**Table 1:** The ten target RSNs, their name^1^ and the involved brain areas (Center of Gravity/Anatomical and Brodmann area (BA)).

RSN	Name ^1)^	x	y	z	Hemisphere	Anatomical Area	BA		mSS	Assigned ICs
1	**Default mode network (DMN)**	19	−20	−10	Right	Parahippocampal Gyrus	35	Hippocampal region	0.56	20/20
		−22	−23	−14	Left	Parahippocampal Gyrus	35	Hippocampal region		
		47	−61	16	Right	Intraparietal sulcus	19	junction of parietal temporal and occipital lobe		
		0	−52	15	Left	Posterior Cingulate	23	Ventral PCC		
		−2	43	11	Left	Anterior Cingulate	32	Dorsal ACC		
		−41	−69	22	Left	Intraparietal sulcus	39	junction of parietal temporal and occipital lobe		
2	**Frontoparietal control network (FPCN)**	43	−57	34	Right	Angular Gyrus	39	part of the temporo-parieto-occipital area	0.40	19/20
		−43	−58	38	Left	Angular Gyrus	39	part of the temporo-parieto-occipital area		
		2	−34	28	Right	Cingulate Gyrus	23	ventral posterior cingulate		
		37	29	33	Right	Middle Frontal Gyrus	9	DLPFC		
		57	−41	−12	Right	Middle Temporal Gyrus	20	higher levels of the ventral stream of visual processing		
		3	20	50	Right	Superior Frontal Gyrus	8	includes FEF		
		−40	35	30	Left	Superior Frontal Gyrus	9	close to FEF		
3	**Frontal attention network (FAN)**	0	20	35	Left	Cingulate Gyrus	32	Dorsal ACC	0.30	19/20
		42	16	6	Right	Insula	13			
		−38	11	7	Left	Insula	13			
		31	37	37	Right	Middle Frontal Gyrus	9	DLPFC		
		−30	37	34	Left	Middle Frontal Gyrus	9	DLPFC		
4	**Left working memory or language network (l-WMN)**	−44	−63	25	Left	Angular Gyrus	39	Part of Wernicke's area	0.39	19/20
		−10	57	6	Left	Medial Frontal Gyrus	10	Anterior prefrontal cortex		
		−46	20	20	Left	Middle Frontal Gyrus	46	DLPFC		
		−59	−30	−8	Left	Middle Temporal Gyrus	21	Auditory processing and language		
		−55	1	−12	Left	Middle Temporal Gyrus	21	Auditory processing and language		
		−11	41	46	Left	Superior Frontal Gyrus	8	Includes FEF		
5	**Right working memory or language network (r-WMN)**	45	33	19	Right	Middle Frontal Gyrus	46	DLPFC	0.39	19/20
		41	−2	49	Right	Middle Frontal Gyrus	6			
		59	−40	−5	Right	Middle Temporal Gyrus	21	Auditory processing and language		
		24	50	39	Right	Superior Frontal Gyrus	8	includes FEF		
		55	−47	28	Right	Supramarginal Gyrus	40	Part of Wernicke's area		
6	**Somato-motor cortex network (SMN)**	36	−28	51	Right	Postcentral Gyrus	3	Primary somatosensory cortex	0.43	15/20
		−49	−12	42	Left	Precentral Gyrus	4	Primary motor cortex		
		−12	−28	52	Left	Medial Frontal Gyrus	6	Premotor cortex		
7	**Auditory cortex network (ACN)**	0,	−14,	45,	Left	Paracentral Lobule	31	Dorsal PCC	0.45	20/20
		−45,	−18,	9,	Left	Superior Temporal Gyrus	41	Primary auditory cortex		
		50,	−17,	10,	Right	Transverse Temporal Gyrus	41	Primary auditory cortex		
8	**Occipital visual network (OVN)**	1	−72	−3	Right	Lingual Gyrus	18	Striate and extrastriate visual cortex (Primary and secondary visual cortex)	0.51	19/20
9	**Ventral visual network (VVN)**	34	−67	−10	Right	Fusiform Gyrus	19	Associative visual cortex (visual “what” pathway)	0.38	16/20
		−36	−67	−12	Left	Fusiform Gyrus	19	Associative visual cortex (visual “what” pathway)		
		31	−53	39	Right	Superior Parietal Lobe	7			
10	**Dorsal visual network (DVN)**	29	−60	41	Right	Superior Parietal Lobule	7	Somatosensory association cortex (ventral visual or “where” pathway)	0.32	18/20
		−30	−58	40	Left	Superior Parietal Lobule	7	Somatosensory association cortex (dorsal visual or “where” pathway)		

For each RSN the mean spatial similarity (mSS) of the subjects' ICs and the Group Component (GC) are reported. The last column indicates the number of subjects that exhibited the respective RSN in an IC and thus contributed to the GC.

Footnote 1) with respect to the nomenclature of RSNs there exist different conventions. Some authors name the RSNs based on the comprised brain areas/lobes (e.g. fronto-parietal-temporal network for RSNs 4&5) others refer to the assumed cognitive functions (e.g. working memory or language network for RSNs 4&5).

### Definition of Frequency Bands

To assemble frequencies with similar topographies we performed a K-means clustering of the t-maps. Most interestingly, the K-means clustering sorted the topographies approximately into the well known standard EEG frequency bands: 1.0Hz<delta≤3.5Hz/3.5Hz<theta1≤6.25Hz/6.25Hz<theta2≤8.2Hz/8.2Hz<alpha1≤10.5Hz/10.5Hz<alpha2≤14.0Hz/14.0Hz<beta1≤18.75Hz/18.75Hz<beta2≤21.88Hz/21.88Hz<beta3≤30.0Hz.

### Covariance Mapping

The Covariance Mapping for the ten selected RSNs revealed specific significant spatial distributions of the spectral scalp field across frequencies (Figure 1).

The randomization test for consistency of the covariance maps across subjects was significant (p<0.05) in 78 out of the total of 80 maps, the two non-significant covariance maps where for theta2 in RSN 5, and for beta3 in RSN 4.

The overall RSN x frequency band TANOVA yielded a significant interaction (p<0.001), indicating that there were significant differences between covariance maps, and that these differences depended on the frequency band. The TANOVAs computed separately for each frequency band were also all significant (p<0.001), indicating that there were consistent differences among the covariance maps of the different RSNs in each frequency band.

T-mapping of the covariance maps indicated that the strongest effects were in the alpha and beta band. Since we normalized the spectral data, covariance maps represent relative spectral power increases or decreases depending on the RSN activity. Positive values indicate a power increase that went along with increased RSN activity, negative values represent a power decrease when the network became active.

- **RSN 1: Default Mode Network (DMN).** DMN activity was found to be associated with increased alpha and beta1 band activity. The covariance and t-maps displayed very similar topographies: Alpha1 increased over central areas with a weak extension to frontal electrodes. Alpha2 showed an increase at posterior occipital electrodes. Beta1 increase was found to be most significant over parietal electrodes. Delta and theta in contrast showed a decrease at fronto-central and parieto-occipital electrodes respectively.
- **RSN 2: Frontoparietal control network (FPCN).** The covariance maps exhibited a strong increase over occipital electrodes bilaterally throughout alpha1, alpha2, beta1 and beta2 while the frontal electrodes especially in alpha2 showed a decrease. Theta on the other hand decreased at central occipital electrodes.
- **RSN 3: Frontal Attention Network (FAN).** The maps found for the FAN were very similar to the maps of the FPCN. Again, the frequencies from alpha1 to beta2 exhibited a topographic distribution indicating anterior-decreases and posterior-increases.
- **RSN 4/5: left and right Working Memory Network (l-WMN/r-WMN).** In left and right WMN - the only two lateralized RSNs - a lateralization could also be observed in the topographies. For the l-WMN the left fronto-temporal electrodes showed decreased alpha and beta1 power while the contralateral occipital and parietal electrodes showed increased power in these frequency bands. For the r-WMN it was vice versa although less pronounced.
- **RSN 6: Somato-Motor Network (SMN).** For this network the covariance and t-maps had topographies with the strongest increases at central electrodes in the delta band whilst the strongest decreases could be observed at centro-parietal electrodes in alpha1 and alpha2. Beta1 and beta2 also showed decreases over centro-parietal areas but was more extended into frontal areas. Beta3 displayed a decrease at central electrodes. For alpha and beta the central decreases went along with surrounding increases.
- **RSN 7: Auditory Cortex Network (ACN).** Similar as for RSN 6 (SMN), the ACN was associated with decreases in alpha1, alpha2, beta1, beta2 and beta3. These spectral power decreases were again most pronounced over central and parietal regions but as compared to the SMN slightly more anterior. The topographies for delta and theta were almost identical to the ones found for SMN.
- **RSN 8/9/10: Occipital Visual Network (OVN) & Ventral Visual Network (VVN) & Dorsal Visual Network (DVN).** The maps for the OVN and the VVN had strong spectral power increases in delta and theta band in the occipital areas while for the DVN the theta increase had its maximum at central electrodes. More interesting were the areas that presented a relative power decrease in alpha and beta bands. For all three visual networks the strongest decreases could be observed at occipital and parietal electrodes. More specifically, the OVN presented with strong negative covariance values with minima at lateral occipital and parietal electrodes. The minima related to the VVN were at similar electrode positions although less pronounced and slightly more lateral. Finally, the VVN displayed a more anteriorized decrease in the alpha as well as the beta bands.

In summary, the first five RSNs associated with higher cognitive functions such as self reflection, attention or working memory and language all displayed a positive association with higher EEG frequency bands while negatively related to delta and theta. In contrast, the last five RSNs that delineate the sensory cortices, i.e. somatomotor, auditory and visual cortices showed positive associations with the lower EEG frequencies but negative association with the higher frequencies. Hence, there seems to be dissociation between sensory networks and networks for higher cognitive functions.

## Discussion

In the present study we investigated the topographic distribution of EEG spectral power in relation to ten fMRI Resting State Networks (RSNs). Fife of our ten RSNs have previously been described in the study of Mantini et al. [19]. DMN, FPCN, OVN, SMC and ACN. The other five networks (FAN, LWMN, RWMN, DVN and VVN) were investigated for the first time with combined EEG-fMRI but are well established in fMRI [10], [11], [20], [27].

However, none of these networks has been investigated regarding their relation to the spatial distribution of the EEG spectral fluctuations. Compared to previous studies that investigated absolute power fluctuations at preselected electrodes or their global field power, there are two important major differences in our work: first, we focussed on the topographic distributions of the spectral power fluctuations and second, we investigated relative fluctuations of spectral power and RSN dynamics. Consequentially, the covariance maps express the relationship between the relative deviations from the mean activities in either modality with detailed spatial information. Information about the spatial relation of EEG spectral fluctuations and RSN dynamics might provide a missing link between the known functional domains of specific RSNs (e.g. attention, vision, motor system) and the existing functional interpretations of localized or global changes in EEG spectra. Moreover, as evident from our results the EEG-RSN covariances showed very different and even opposite behavior depending on electrode location, which might partly explain why there were divergent results in some of the of former studies.

### General observations

The three main findings were (i) that each of the ten investigated RSNs showed a specific, frequency dependent distribution of EEG spectral power changes, (ii) that networks for higher cognitive functions showed an inverted relationship compared to primary sensory networks and (iii) that the topographic maps across all RSNs and all frequencies clustered into the standard EEG frequency bands.

There was a statistically significant consistency of the effects for almost all RSNs and frequency bands. Hence, although the resting state is apparently only vaguely defined, it has common norms across healthy subjects. Indeed, a disruption of these common processes has been observed in different mental diseases ([13] fMRI; [3] EEG).

The spatial patterns of the covariance maps in the different frequency bands and for the different RSNs are in good accordance with EEG topographies reported in the literature [28], [29]). While the delta and theta band bare their strongest effects in frontocentral areas, alpha1 had its main effects distributed in centro-posterior regions and alpha2 predominately in occipital areas. Finally, beta showed the most significant covariances anterior to alpha but posterior to delta and theta.

Besides the accordance of the topographies with the known EEG norms, there is further consistency of our findings with EEG-fMRI literature. The negative association of the alpha band with visual areas (OVN, VVN & DVN) was first reported by Goldman [15] and repeatedly replicated [16], [19]. Additionally, alpha (and beta) was inversely related to the SMN and the ACN [19], [30]. A positive relationship of alpha and beta power fluctuations was reported for the DMN [17], [19] whereas frontal theta correlated negatively with the DMN [22].

A third consistency was that the t-maps clustered into the standard EEG frequency bands. Notably, these maps held no information about the frequency bin from which they were calculated. This finding suggests that the EEG frequency bands indeed can be seen as electrophysiological signatures of functional networks as often argued in the EEG research.

From these findings we can derive several hypotheses: For a given RSN, the different frequency bands had different covariance maps implying that the different bands serve separate subfunctions of the same network. Other combined EEG-fMRI studies support this hypothesis demonstrating that one RSN associates with several frequencies [19] and that only specific subregions of a RSN correlate with spectral fluctuations of one frequency band [17]. Furthermore, within one frequency band, the covariance maps differed for the different RSNs. This observation implies that distinct cognitive functions are most likely reflected by EEG activity that may occur at the same frequency but have different spatial distributions [31]. This is conceivable under the consideration that neurons respectively neuronal assemblies have a limited range of oscillatory properties. Hence, the coordination of distinct cognitive processes is achieved by synchronizing the activity of distributed brain areas within and across frequencies reflected in the EEG as spatially distinct rhythms associated to different RSNs.

In sum, our data suggests that EEG frequency bands reflect topographically organized rhythms related to subfunctions of known neurocognitive networks. And we provide novel and detailed spatial knowledge about the relationship between EEG spectral data and RSN dynamics.

### Specific observations

Besides these general observations our analyses also yielded some specific results. The covariance maps (Figure 1 right) suggest an inverse relation between slow (delta, theta) and fast (alpha, beta) EEG rhythms [28], [32]. While this anticorrelation of slow and fast rhythms was present across all RSNs, the polarity of the maps was inverted between the first five RSNs and the last fives. Interestingly, the first five RSN (DMN, FPCN, FAN & left and right WMN) are involved in higher cognitive processes such as self-referential, attentional or memory processes while the other five RSNs all represent primary or secondary sensory networks (SMN, ACN, OVN, VVN & DVN) suggesting that brain resources are dynamically reallocated between attentional or self-referential networks and to the sensory processing units [33]. In addition, this competition for resources seems to be related to slow and fast EEG rhythms. In the following section we will briefly discuss the cognitive processes that are associated with the frequency bands and the respective RSNs.

#### Higher cognitive networks

In the networks thought to be involved in attentional, self-referential and memory processes, alpha and beta power increased with increasing activity. This is in line with current views that alpha plays a role in attentional and working memory processes (for a review see [34], [35]). While the lower alpha band was proposed to be involved in attention, the upper alpha band mainly was associated with working memory. Furthermore, it was repeatedly demonstrated that increased activity of the DMN is positively correlated to alpha power [17], [18], [19] and that DMN activity is associated to lapses in attention [36]. In addition, it has been argued that cross-frequency synchronization between alpha and beta might be important for the coordination of attention [37], [38]. This cross-synchronization might be a base for the topographic similarities between alpha and beta covariance maps in the present study.

While the DMN, the FPCN and the FAN displayed symmetrical covariance maps, the two WMNs presented a lateralized alpha decrease at anterior-temporal areas over the networks' hemispheres, while on the contralateral side an alpha and a weak theta increase could be observed. This may indicate the often observed alpha suppression/surround synchronization in experimental settings with working memory involvement [34], [39].

Besides alpha and beta, another commonly described EEG rhythm, the frontal midline theta, has been observed during various cognitive tasks that require attention or memory [40], and it was found to correlate negatively with areas comprised by the DMN and the WMNs [22], [41]. This negative correlation of medial frontal electrodes in the theta frequency was also evident in the present study.

Considering these facts our findings are in accordance with the present theory of EEG spectral correlates of attentional and memory processes. Our results further support the hypothesis that alpha and beta activity modulates and focuses attention and mediates working memory functions. Moreover, the distinct networks involved in those processes might be coordinated by regional fluctuations across frequency bands ranging from theta, through alpha to beta.

#### Sensory networks

In contrast to the higher cognitive networks, the sensory networks showed a suppression of alpha and beta power during increased network activity. This is concordant with previous publications showing an inverse relationship between alpha respectively beta power with BOLD signal fluctuations in sensory networks [15], [16], [19], [30]. But in addition to those previous studies we provide the topographic patterns of the spectral power fluctuations and demonstrate a selective suppression of alpha and beta power over the respective sensory cortices constituting the distinct sensory RSNs.

Thus, the SMN is expectedly associated with the so called EEG rolandic alpha [42], [43] and beta [44] rhythms, located at central/parietal electrodes whereas for the visual networks an association to the posterior/occipital alpha rhythm is presumed. This is clearly supported by the topography of the alpha-band covariance maps of the SMN [30] and of the three visual networks (OVN, VVN & DVN). While the covariance maps of the SMN matched the distribution of the rolandic alpha *(and beta)*, the three visual networks had negative covariance values predominately at occipital and posterior electrodes. The OVN showed a typical occipital alpha rhythm distribution throughout alpha and beta. Furthermore, the negative relationship between alpha and OVN was consistent with the works of Goldman [15], Laufs [16] and Mantini [19]. The maps found for the VVN were very similar but slightly more lateral, matching the VVN's more lateral localization. In contrast, the DVN was associated with covariance maps extending into more anterior electrodes. Hence, although the topographies across these three RSNs show similarities, there were also divergences that most likely depict the spatial differences of the underlying RSNs.

Further support for the view that slight differences in the covariance maps reflect the distinct spatial organizations of the RSNs is given by the comparison of the SMN and the ACN. Both RSNs have a similar spatial distribution of their nodes including a medial area and two lateral regions. While in the SMN the lateral regions cover the somatomotor cortices, in the ACN the two superior temporal gyri are involved. Functionally, these regions serve completely different purposes but in terms of EEG they might generate similar electrical fields as evident in the covariance maps.

### Outlook

The methodology presented in our study systematically linked EEG variations to the dynamics of RSNs. Both in EEG and fMRI, it was repeatedly demonstrated that deviations from the resting state configuration either represent on- or offset of cognitive processes, or indicate pathopysiological alterations of the normal baseline state. As an example, schizophrenic patients on the one side present with altered behavioral performance during different cognitive tasks, especially during working memory tasks. On the other side, it is well established that schizophrenic patients show deviant EEG topographies during a task condition but also during rest [45]. More recently, altered RSNs have been reported for schizophrenia patients as compared to matched healthy controls (DMN, LWMN [46]) suggesting that their behavioral deficits are related to an aberrant baseline state.

Furthermore, a recent paper has demonstrated correspondences of RSNs with transient sub-second states of synchronized EEG brain states (microstates, [31]) and we have previously shown fMRI correlates of EEG synchronization in the frequency domain [17]. It will thus be interesting to explore the relationship of network formation on different time-scales as seen by fMRI, and frequency and time domain EEG. This might further help to understand the complex relation between functional networks assessed by fMRI and the functional states identified in EEG.

### Limitations

Although we demonstrated a significant spatial relationship between RSNs and EEG frequency fluctuations the presented covariance maps do not provide direct information about the intracerebral location of the involved EEG sources, and does not allow assigning a specific EEG rhythm to subcomponents of a RSN. This assignment could eventually be achieved by estimating EEG inverse solutions. However, EEG inverse solutions are heavily model-dependent, which may again introduce uncertainties in the results that we wanted to avoid given the already considerable complexity of our results and the controversy in the current literature. Moreover, the resolution of EEG inverse solutions decays with depth [47], such that the proper estimation of activity in key regions like deep midline regions of the DMN introduced even more uncertainties.

Furthermore, the resting state is defined as a state of relaxed wakefulness when subjects have their eyes closed and are instructed to refrain from any structured thoughts. These instructions are vague and the resting state remains a rather uncontrollable and unconstrained condition with high variability between subjects. Nevertheless, as argued above, there must be some common processes besides all the likely differences between individuals.

## Methods

### Ethics Statement

The study has been approved by the local ethics committee (“Kantonale Ethikkimmission Bern”). Subjects were recruited among university students and gave their written informed consent.

### Subjects

We measured 20 healthy young subjects (10 female; mean age ± SD: 26±3 years). All subjects were measured in the morning between 8 am and 11 am. They refrained from caffeine, nicotine and alcohol at least 10 hours prior to the experiment. Any contraindications against MRI, use of psychoactive medication or illegal drugs as well as neurologic or psychiatric history were exclusion criteria.

### MRI data acquisition

We used a 3T Siemens Magnetom Trio MR Scanner (Siemens, Erlangen, Germany). The functional T2* weighted MR images were acquired with an echo planar imaging (EPI) sequence. The characteristics of this sequence were: 252 volumes, 32 slices, 3×3×3 mm^3^, gap thickness 0.75 mm, matrix size 64×64, FOV 192×192 mm^2^, TR/TE 1980ms/30ms.

A structural T1 weighted sequence was recorded after the simultaneous EEG-fMRI and removal of the EEG cap. The parameters of this fast low angle shot (FLASH) sequence were: 176 sagittal slices, slice thickness 1.0 mm, FOV 256×256 mm^2^, TR/TE 2300ms/3.93ms.

### EEG data acquisition

The EEG was acquired with a 96 channel MR compatible system from Brain Products (Gilching, Germany/input range: 16.3mV, resolution: 0.25µV). Four channels were used to record the electrocardiogram (ECG; 2 electrodes below the clavicles) and the electrooculogram (EOG; 2 electrodes below the eyes). The clock of the recording computer was synchronized to the clock of the MR system (10 kHz refresh rate). Each MR scan volume was automatically marked in the EEG data. The EEG was bandpass filtered between 0.1 Hz and 250 Hz and sampled with 5 kHz. Impedances between the electrodes and the subject's scalp were kept below 50 kΩ.

#### Combined recording

An app. 9 min simultaneous EEG-fMRI recording was performed while the subjects were in a state of relaxed wakefulness referred to as the resting state. Subjects lay with their eyes closed inside the scanner and were instructed not to think anything in particular and especially to try to relax while not falling asleep.

### fMRI Data preprocessing and data extraction

The MR datasets were processed in BrainVoyagerQX (Version 1.10.2, Brain Innovation, Maastricht, The Netherlands). Preprocessing of the functional images included slice scan time correction, linear trend removal, 3D motion detection and correction and spatial smoothing with a Gaussian Kernel (FWHM 8mm). The functional images were co-registered to the anatomical images, rotated into the anterior-posterior commissural plane and normalized into standard Talairach space [48]. The fMRI timecourses were z-transformed (mean = 0, SD = 1) voxelwise.

Resting State Networks (RSNs) were identified using an Independent Component Analysis (ICA) that decomposed the individual's fMRI into 30 statistically independent components (ICs), each delineating groups of voxels that exhibited synchronous temporal fluctuations. Each IC consisted of a spatial map and its mean network dynamics [49], [50]. For each subject, the resulting ICs were visually inspected and assigned to ten previously described RSNs (Table 1) [10], [11], [24]. The assignment of an IC to a RSN was based on its spatial similarity and IC characteristics [25]. To check for the consistency of the assignment across subjects, we calculated a group component (GC) using a t-test across the individuals' spatial ICs for each RSN. Each GC consisted of a 3D matrix (x y z coordinates) of t-scores (consistency of each voxel in the GC across the 20 subjects). For display the threshold was set at p<0.005 corrected for false positives with a spatial extend threshold at alpha 0.05. Additionally, we computed the spatial similarity of each subject's IC to their specific GC and tabulated the mean similarities (according to [26]).

Using Matlab routines, we back-projected the individual z-transformed fMRI BOLD data onto the obtained group components, yielding a timecourse of relative activity for each RSN and subject based on common spatial templates. Back-projection denotes using the GC map as a weighting matrix for the individual voxel timecourses (i.e. computing the dot-product of the GC map with all momentary BOLD images). By deriving the RSN dynamics of the individual BOLD signals from a common average RSN spatial template, we prevented that individual differences in the spatial distribution of the identified RSN would confound the further analysis (ICA decomposition is based on a random starting matrix that yields slight run-by-run differences, furthermore the a priori selected order of the ICA might lead to fragmented or insufficiently decomposed networks in different subjects [24], [51], [52]). The resulting RSN timecourses were normalized for unit variance across RSNs for each volume.

### EEG preprocessing and data extraction

All EEG preprocessing was performed in Vision Analyzer (Version 1.05.0005; Brain Products, Gilching, Germany). First, the EEG was corrected for artifacts. This included scan-pulse artifact correction by average artifact subtraction (AAS) [53] and cardio-ballistic actifact (CBA) correction using Independent Component Analysis (ICA) [17], [54]. For a detailed description of the ICA based CBA correction see Jann et al. [17]. Thereafter, the EEG was visually inspected and marked for additional artifacts (i.e. residual scanner artifacts and motion artifacts). Electrode channels exhibiting excessive artifacts were interpolated using a spherical spline interpolation. Finally, the data was bandpass filtered (1–30Hz), downsampled to 100Hz and recomputed to average reference.

The EEG was segmented into epochs of 256 datapoints in timewindows 6560ms – 4010ms before each MR scan onset, excluding epochs containing artifacts. This timewindow accounts for the typical delay of the hemodynamic response (HR) related to neuronal activity. Each epoch was FFT transformed (resolution 0.39 Hz, Hanning window 10%), yielding (in the 1–30Hz band) 75 frequency bins. Paralleling the analysis of the fMRI RSN data, the mean spectral amplitude across epochs was removed in each channel and frequency, and the data was normalized in each epoch and frequency bin to have unit variance across channels.

### Combination: Covariance Mapping of the fMRI GCs (RSNs) and the EEG

The covariance between the normalized individual datasets extracted from the EEG respectively fMRI were calculated similar to the approach presented in Koenig et al. [23]. For each subject, the dynamics of the normalized EEG spectral amplitude at each electrode and frequency bin were dot-multiplied with the dynamics of each RSN, resulting in a data matrix of 20 (subjects)×10 (RSNs)×75 (frequency steps)×92 (electrodes) covariance values.

### Statistics

The following statistical analysis was designed to reduce the size of the result space by averaging the covariance values across frequency, to apply tests for the consistency of the result across subjects and to test for systematic effects of frequency band and RSN while protecting the final results against false positives due to multiple testing. The analyses are briefly outlined below, followed by a more detailed description of the separate steps and tests.

Since all preprocessed data had been centered to have zero-mean across epochs, we chose to use independent one-sample t-test values (against zero) across subjects, so-called t-maps, to visualize the consistency across subjects. These t-maps were not used to infer the probability of null-hypotheses, but merely as standardized indices of signal-to-noise ratios across subjects. Based on these t-maps, we reduced the redundancy of the data in frequency by a cluster analysis that collapsed the data into a few frequency bands. The across-subject consistency of the covariance maps of each RSN in each band was then assessed.

Furthermore, it was assessed whether there were significant differences of the encountered covariance maps as function of RSN and frequency. This was tested with a two-factorial topographic analysis of variance (TANOVA;[55], [56]) with the factors RSN and frequency band as repeated measures. This overall TANOVA was followed by post-hoc TANOVAs testing for differences of covariance maps among RSNs separately for each frequency band.

### K-Means clustering to identify boarders of frequency bands

Firstly, we attempted to identify the borders of the frequency bands employing a k-means clustering algorithm. As data vectors, the clustering algorithm used the t-maps of all 10 RSNs (920 values), distances between data vectors were measured using the Euclidian distance. Since the primary aim of the cluster analysis was to establish the exact borders of frequency bands in our data based on the t-maps, we attempted to obtain a similar amount of clusters as previously used frequency bands by increasing the cluster number until an apparently optimal match was found.

### Topographic consistency testing to test the significance of the topographies

Next, mean covariance maps within the identified frequency bands were computed for each subject, and mean and t-maps across subjects were computed again. Since these t-maps were exploratory in nature, we applied global randomization tests (the topographic consistency test [55], [57]) for the consistency of the covariance maps across subjects as function of frequency band and RSN. The rationale of this test is the following: Under the null hypothesis, the individual covariance values at a given electrode are expected to have a distribution with zero mean. Means across subjects differing from zero therefore indicate some deviation from the null-hypothesis. To have an overall index of deviations from zero across all channels, the root-mean-square (RMS) across channels of the average covariance was computed. In a next step, this RMS value had to be tested for its probability of having been obtained while the null-hypothesis was true. This probability was estimated using randomization techniques: In each subject, the covariance values were randomly shuffled among electrodes, producing datasets with the same overall variance as the original data, but with a possible common spatial structure across subjects destroyed. 5000 such datasets were produced, and in each of these datasets, the RMS was computed as outlined above. The probability that a covariance map has been obtained by change (significance) is then defined as the percentage of the 5000 randomly obtained RMS values cases that were larger than the RMS value obtained in the un-shuffled data. This randomization test was applied to the covariance maps obtained in each frequency-band and with each RSN.

### TANOVAs: significant effects of the results between frequency bands and RSNs

The next analysis tested whether there were consistent differences between the covariance maps of the different RSNs. For this purpose we submitted the individual covariance maps of all RSN to a topographic analysis of variance (TANOVA, [55], [56]). TANOVAs assess differences between maps using a global, across all electrodes difference measure and test for the significance of the observed differences using randomization techniques. Contrary to other methods, a pre-selection of electrodes and/or correcting for multiple testing across electrodes is thus not necessary. We computed an overall, two-factorial TANOVA with the covariance maps of all RSNs and all frequency bands, treating frequency and RSN as repeated measures, and we computed TANOVAs separately for each frequency band, with RSNs as repeated measures. All TANOVAs were based on 5000 randomization runs.
